# PDE9A is expressed in the inner retina and contributes to the normal shape of the photopic ERG waveform

**DOI:** 10.3389/fnmol.2014.00060

**Published:** 2014-06-27

**Authors:** Anuradha Dhingra, Shanti R. Tummala, Arkady Lyubarsky, Noga Vardi

**Affiliations:** ^1^Retina Lab, Department of Neuroscience, University of PennsylvaniaPhiladelphia, PA, USA; ^2^Department of Ophthalmology, University of PennsylvaniaPhiladelphia, PA, USA

**Keywords:** ERG, serial inhibition, amacrine cells, cyclic GMP, cone pathways, ciliary body

## Abstract

The ubiquitous second messenger cGMP is synthesized by guanylyl cyclase and hydrolyzed by phosphodiesterase (PDE). cGMP mediates numerous signaling pathways in multiple tissues. In the retina, cGMP regulates signaling in nearly every cell class including photoreceptors, bipolar cells, amacrine cells, and ganglion cells. In order to understand the specific role of cGMP and its regulating enzymes in different cell types, it is first necessary to localize these components and dissect their influence on the circuits. Here we tested the contribution of PDE9A to retinal processing by recording the electroretinograms (ERG) of *PDE9A*^™/™^ (KO) mice and by localizing the enzyme. We found that while the scotopic ERG of KO was the same as that of wild type (WT) in both amplitude and kinetics, the photopic ERG was greatly affected. The greatest effect was on the recovery of the b-wave; the falling phase and the b-wave duration were significantly longer in the KO mice for all photopic stimuli (UV, green, or saturating white flashes). The rising phase was slower in KO than in WT for UV and green stimuli. For certain stimuli, amplitudes of both the a- and b-waves were smaller than in WT. Using *Lac-Z* expression in KO retinas as a reporter for PDE9A expression pattern, we found that PDE9A is localized to GABA-positive and GABA-negative amacrine cells, and likely also to certain types of ganglion cells. Our results indicate that PDE9A, by controlling the level of cGMP, modulates inhibitory processes within the cone pathway. We speculate that these circuits involve NO/cGMP signaling pathways.

## Introduction

The ubiquitous second messenger cGMP, which is synthesized by guanylyl cyclases (GC) and hydrolyzed by phospodiesterases (PDE), controls a variety of processes from smooth muscle cell contraction in the vasculature to signaling in the central nervous system (Beavo, 1995; Polson and Strada, 1996; Juilfs et al., 1999). cGMP typically accomplishes these functions either by gating cyclic nucleotide-gated (CNG) channels or by activating cGMP-dependent kinases and phosphatases. In the retina, cGMP regulates nearly every step of signal processing. At the first step of retinal processing, rod and cone photoreceptors use cGMP to gate CNG channels. In these cells, light activates PDE6, which hydrolyzes cGMP; this closes the CNG channels and the cells hyperpolarize (Lamb and Pugh, 1992; Hsu and Molday, 1993; Yau, 1994). At the next step of retinal processing, ON bipolar cells (rod bipolar that mediate night vision and ON cone bipolar cells that signal light increments in daylight vision) appear to use cGMP, but its function is still unclear. cGMP was initially thought to gate a CNG channel similar to photoreceptors (Nawy and Jahr, 1990; Shiells and Falk, 1990, 1992; de la Villa et al., 1995). It is now known that ON bipolar cells use the non-selective cation channel TRPM1, which is not gated by cGMP (Morgans et al., 2009; Koike et al., 2010). Instead, some evidence suggests that cGMP potentiates the light response at low light intensities (Nawy, 1999; Shiells and Falk, 2002; Snellman and Nawy, 2004). At the next stage of visual processing, some third order neurons (including certain types of amacrine and ganglion cells) modulate cGMP, often in response to NO (Ahmad et al., 1994; Gotzes et al., 1998; Chun et al., 1999; Blute et al., 2003). The NO/cGMP pathway is also critical for modulating coupling conductance between the AII amacrine cells and ON cone bipolar cells (Mills and Massey, 1995). The exact role and mechanism of cGMP regulation in second and third order neurons is not clear. In order to better understand the role of cGMP in different steps of processing, and especially in ON bipolar cells, it is important to localize PDE enzymes in retina. To this end, using our ON bipolar cell cDNA library, we identified a PDE isoform *PDE9A* and determined that these cells express the transcript for this isoform (Dhingra et al., 2008). Moreover analysis of the retinal transcriptome database (Siegert et al., 2009) showed that several amacrine and ganglion cell types also express *PDE9A* transcripts. Because PDE9A is expressed predominately in neurons (van Staveren and Markerink-van Ittersum, 2005), and hydrolyses cGMP with a very low Km (Fisher et al., 1998), it likely contributes to retinal function. Here, using *PDE9A*^−/−^ (KO) mouse, we report that PDE9A contributes to the normal kinetics of the photopic electroretinogram (ERG). PDE9A is localized to amacrine cells, ganglion cells, and to the ciliary body, but not ON bipolar cells. We therefore conclude that inner retinal expression of PDE9A in amacrine cells contributes to response kinetics of the ERG b-wave.

## Materials and methods

### Generation of *PDE9A* knockout mice

*PDE9A*^−/−^ (KO) mice were obtained from Pfizer (Menniti et al., 2008) and were initially generated by Deltagen (San Mateo, CA). Briefly, a targeting construct with neomycin resistance (neo) and bacterial *LacZ* (coding for β-galactosidase or β-gal) genes was designed to disrupt the *PDE9A* gene (Accession # AF031147) in ES cells by homologous recombination. The resultant allele had a replacement of part of exon 11/12 of *PDE9A* with neo-*LacZ* cassette that shifted the downstream sequence out of the open reading frame. The ES cells were injected into blastocysts and the resulting chimeras were tested for germ line transmission. The following primers were used for genotyping:

GS (E) (CACAGATGATGTACAGTATGGTCTGG);

GS (T,E) (TGCAGTCATCAGGACCAAGATGTCC); and

Neo (T) (GACGAGTTCTTCTGAGGGGATCGATC).

Genotyping was performed on tail DNA using a multiplex PCR reaction: GS (T, E) + Neo (T) for targeted allele (expected size 599 bp); GS (E) + GS (T, E) for endogenous allele (expected size 256 bp). KO mice were bred to homogenicity by multiple back-crossings (>6) with C57BL6/J mates. Mouse colonies were maintained by breeding KO mice; no specific behavioral phenotypes were observed in these mice. Age matched C57BL6/J (WT) were purchased from Jackson Labs (Bar Harbor, Maine). All experiments were done in compliance with federal regulations and the protocol was reviewed and approved by the Institutional Animal Care and Use Committee of the University of Pennsylvania. Both male and female mice were used for all experiments.

### Electroretinogram

The ERG recording setup and methods have been described elaborately (Lyubarsky et al., 1999, 2000; Ng et al., 2010). In short, mice were dark-adapted, deeply anesthetized intraperitoneally under dim light with ketamine/xylazine/urethane (20, 8, and 800 μg/gm bodyweight, respectively) and were placed on a platform maintained at 37–38°C. Pupils were dilated with 1% tropicamide saline solution (Mydriacyl, Alconox). A platinum electrode was inserted into the mouth to serve as a reference and ground electrode, and another platinum electrode was placed on the cornea. Mice were placed inside a light-proof Ganzfield Faraday cage. ERG recordings from KO and age matched WT mice were performed on the same day under identical settings and conditions. Light stimuli were either 4 ms flashes produced by a light-emitting diode (LED) stimulator or <1 ms flashes produced by a Xenon tube delivered in the Ganzfeld (Espion Electrophysiology System; Diagnosys). For scotopic ERG, intensities of light flashes were converted to the estimated number of photoisomerizations (R^*^) per rod as described previously (Lyubarsky et al., 2004). For photopic ERG, light intensity was converted to number of photons per μm^2^ at the cornea as described previously (Lyubarsky et al., 1999, 2000). For photopic stimuli, a rod-suppressing step of light (30 scot cd m^−2^) was given every 5.5 s and a light flash was superimposed on it 2 s after the onset of the step. ERGs were recorded from both eyes using differential amplifiers with a bandwidth of 0.1 Hz–1 kHz, and a sampling interval of 1 ms. Depending on the signal-to-noise ratio, each record was an average of 3–25 individual trials.

The various parameters characterizing the ERG waveform were determined using a user-defined MATLAB® (Mathworks, MA) program. The amplitude of the b-wave was quantified by subtracting the peak of the a-wave from the peak of the b-wave. The functional characteristics of phototransduction in photoreceptors in WT and KO animals were determined from the ERG a-wave by fitting the rising phase of the a-wave with the transduction cascade activation model (Breton et al., 1994; Lyubarsky and Pugh, 1996) as follows:

(1) $$F(t)=\exp[-0.5\Phi A{\left(t-t_{eff}\right)}^{2}],$$

where *F(t)* is the a-wave amplitude at time *t* after a brief flash normalized to the saturated amplitude; Φ is the number of photoisomerizations per rod; *t_eff_* a brief delay; *A* is the amplification constant whose value is estimated by the best fit. ERG data presented in this paper were performed on 12 WT and 14 KO mice, but occasional records were too noisy and were not considered. Values of the measured response parameters for the left and right eyes were averaged and treated as a single data point. The number of animals or data points acquired for each stimulus condition is specified in the Results section. All data values in the figures are presented as mean ± s.e.m. Unless stated otherwise, statistical comparison of a parameter between groups of WT and KO was performed using two-tailed Student’s *t*-test. A *p*-value of less than 0.05 (*p* < 0.05) was considered significant and *p* < 0.01 was considered highly significant.

### β-galactosidase assay

Mice were deeply anesthetized with an intraperitoneal injection of ketamine and xylazine (100 and 10 μg/gm bodyweight). Eyes were enucleated and incised just below the limbus. Eyeballs were fixed in 4% paraformaldehyde for 10 min, and rinsed in 0.1 M phosphate buffer. For cryosections, eyeballs were cryoprotected by soaking overnight at 4°C in 0.1 M phosphate buffer containing 30% sucrose and embedded in a mixture of two parts 20% sucrose in phosphate buffer and one part optimal cutting temperature compound (Tissue Tek, Electron Microscopy Sciences, Hatfield, PA, USA). Radial sections (15–20 μm) were cut on a cryostat (Leica) at −20°C and collected on superfrost plus slides (Fisher Scientific, Pittsburgh, PA, USA). β-galactosidase expression was detected enzymatically using X-gal (5-bromo-3-indoyl-b-D-galactopyranoside stock solution) as substrate. Briefly, whole mount or retinal sections were pre-incubated in a detergent buffer (0.02% Nonidet P-40, 0.01% Sodium Deoxycholate, and 2 mM MgCl_2_ in 0.1 M phosphate buffer, pH 7.3) for about 10 mins at room temperature (RT), incubated for 2–4 days in staining buffer (0.02% Nonidet P-40, 0.01% Sodium Deoxycholate, 5 mM Potassium Ferricyanide, 5 mM Potassium Ferrocyanide, and 2 mM MgCl_2_ in 0.1 M phosphate buffer, pH 7.3 containing 1–1.5 mg/ml X-gal) and finally rinsed twice in detergent buffer (5 min each).

### Immunofluorescence

Sections were permeabilized and blocked with 10% normal goat serum, 5% sucrose and 0.5% Triton X-100 in phosphate buffer for 1 h at RT. Sections were incubated in primary antibodies (diluted in blocking solution) at 4°C overnight or occasionally for 3 days. Primary antibodies were washed 3 times in 5% sucrose in phosphate buffer at RT. Sections were then incubated in secondary antibodies at RT for 3 h, washed, mounted with Vectashield mounting medium (Vector Laboratories, Burlingame, CA, USA) and coverslipped. To identify X-gal-stained cells in the inner retina, some retinal sections were first stained for GABA and then processed for X-gal staining, and others were first processed for X-gal staining, and then immunostained for GABA. For double staining on the whole mount, we first stained for GABA overnight and then with x-gal for at least 3 days; after imaging, the retinas were also stained with DAPI. To avoid collapse of retinal layers, retinas were mounted between number 0 cover slips, which served to support the coverslip. The blue staining corresponding to the sites of β-galactosidase activity and the fluorescent stainings were examined with a conventional microscope (Nikon Eclipse TE-300 microscope; Nikon Inc., Melville, NY, USA).

#### Antibodies

Rabbit anti-PDE9A (1:100–500, GTX14625 from Genetex, Irvine, CA); rabbit anti-PDE9A (1:100, PD9A-101AP from FabGennix, Frisco, TX); guinea-pig anti-GABA (1:1000, AB175, Chemicon International, Temecula, CA); mouse anti-β-gal antibodies (a monoclonal; 1:200, Promega, Madison, WI); and chicken anti-β-gal (1:500, Aves labs Inc., Tigard, Oregon).

## Results

### Absence of PDE9A does not affect the scotopic ERG, but decreases the amplitude of the rod/cone-generated ERG a-wave

**Figure 1:**
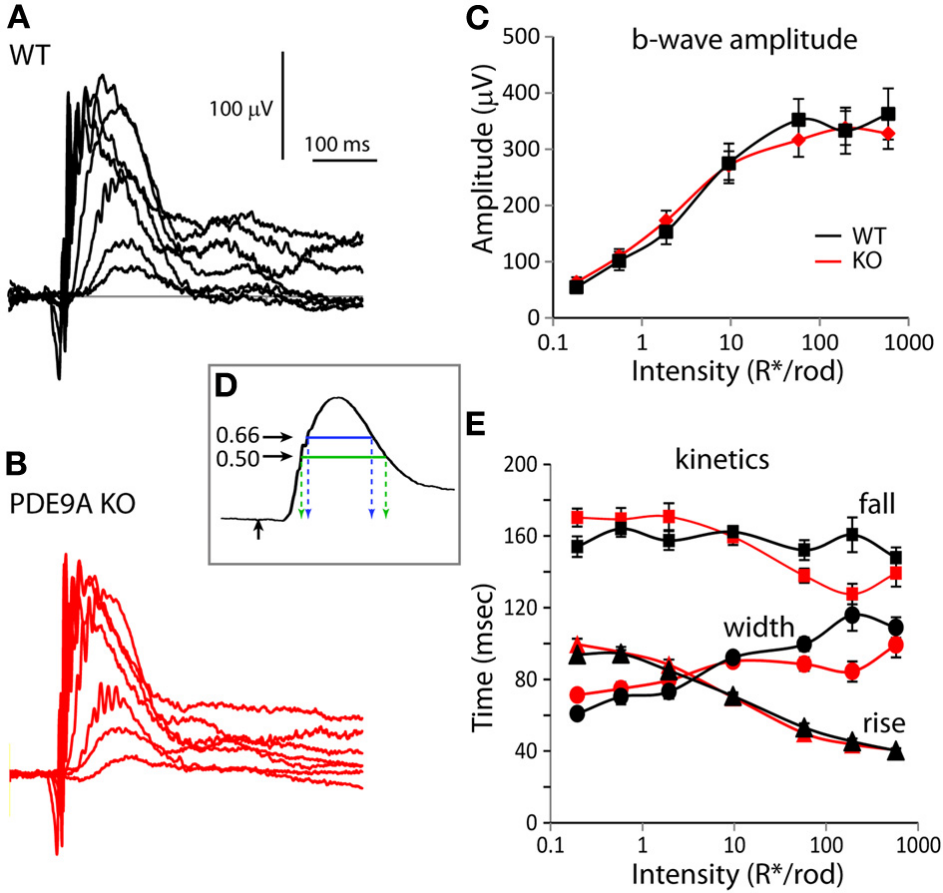
**PDE9A does not contribute to the ERG under scotopic conditions**. For all figures, WT is drawn in black and KO in red. Also for all figures, data are plotted as average ±s.e.m. When the s.e.m. cannot be seen, it is smaller than the symbol. **(A,B)** Representative responses to increasing light intensities (0.2– 600 R^*^/rod) from age matched WT **(A)** and *PDE9A* KO **(B)** retinas. **(C)** Average b-wave amplitudes vs. flash intensity for WT (black squares; 16–22 records) and KO (red diamonds; 18–28 records) mice. **(D)** Illustration of the parameters used to characterize the kinetics of the b-wave. Stimulus onset is designated by an upward black arrow, 66% of the peak amplitude is indicated by the blue line and 50% by the green line. Time taken by the b-wave to rise from the stimulus onset to 66% of its peak amplitude (left dashed blue arrow) or to 50% of its amplitude (left dashed green arrow) were used for comparison of its kinetics. Time taken by the b-wave to fall to 66 or 50% of its peak amplitude is indicated by the right blue and green arrows. **(E)** Times taken by the b-wave to rise (triangles) and fall (squares) to 66% of its amplitude, and the duration between them (width, circles) at the measured intensities.

To determine if PDE9A contributes to visual processing, we recorded ERGs from WT and *PDE9A* KO mice. We first presented a series of scotopic flash intensities under dark-adapted conditions and measured the amplitude of the b-wave. There was no significant difference in the ERG b-wave amplitude between KO and WT (Figures 1A–C). To assess the response kinetics, we measured the time it took the b-wave to rise to 66% of its peak amplitude, the time it took to fall to 66% of its peak amplitude, and the 66% maximum width (as in Figure 1D). We also analyzed the half width; however, because responses of dark-adapted mice at high intensities often do not return to baseline in the sampled duration of 500 ms, this measure is less reliable and is not reported here. Under scotopic conditions, the kinetic parameters of KO and WT ERGs were similar (Figure 1E).

**Figure 2:**
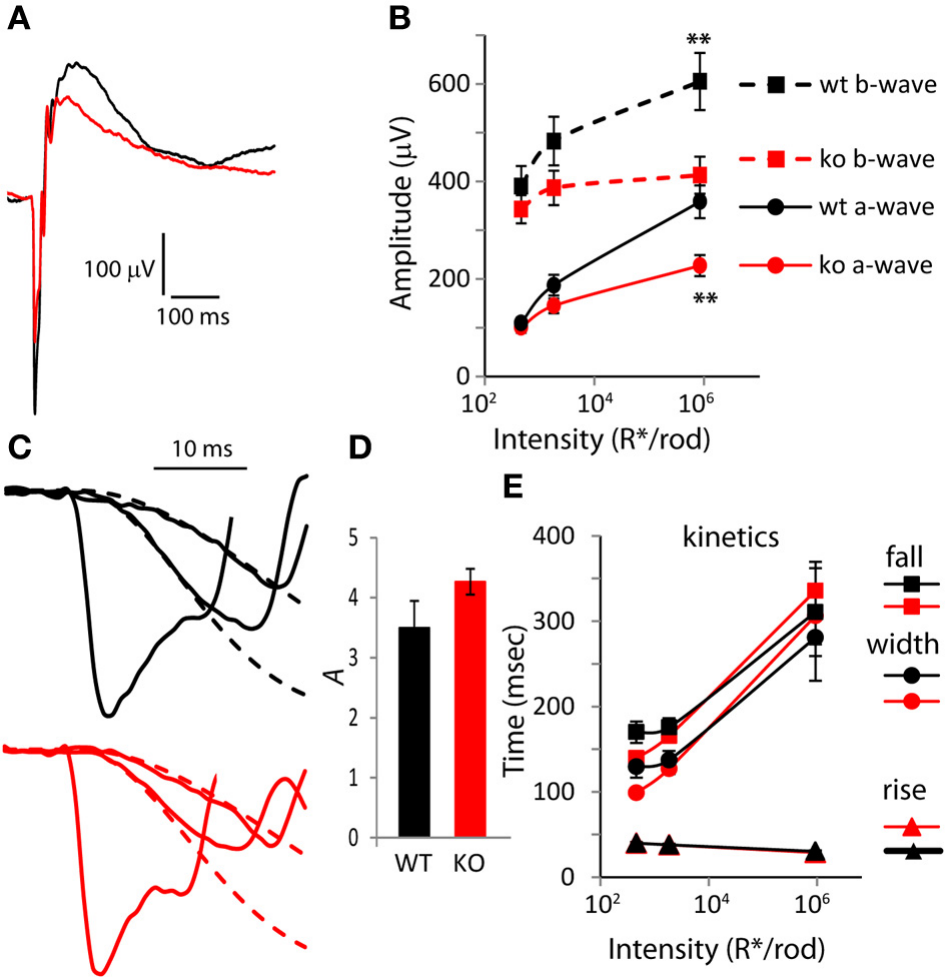
**The amplitude of the a-wave is smaller in *PDE9A* KO but its kinetics is largely unaffected. (A)** Average response from WT and KO retinas to a rod saturating flash (~10^6^ R^*^/rod). **(B)** Average a-wave and b-wave amplitudes (dark-adapted, rod and cone generated responses). Both a-wave and b-wave amplitudes in KO (*n* = 14 mice for all intensities) are significantly different from WT (*n* = 12) at the two highest intensities. Unless otherwise stated, for all figures, “*” designates *p* < 0.05 and ^**^p < 0.01. **(C)** Expanded ERG responses from a KO and WT animal at the flash intensities in **(B)** showing the a-waves and their Lamb and Pugh curve fits (red and black dashed lines for KO and WT, respectively). The Lamb and Pugh equation was fit to the average a-wave from both eyes of the animal to estimate the amplification constant. **(D)** The Lamb and Pugh amplification constants as estimated by regression in WT and KO animals are similar. **(E)** The time taken by the b-wave to rise and fall to 66% of its amplitude and the duration (width). For the highest intensity, the b-wave fell to 66% of its amplitude only in 50% of WT and in 82% of KO.

Next, to determine the possible contribution of PDE9A to the a-wave, we presented strong saturating flashes that stimulate both rods and cones (Figure 2A). In response to the strongest flash (*I* = 9.3 × 10^5^ R^*^/rod), both the a- and b-wave amplitudes were significantly lower in KO than in WT. The a-wave saturating amplitude in KO (227 μV) was 36% lower than in WT (357 μV; *p* = 0.002), and the b-wave saturating amplitude of KO was 30% lower (411 μV in KO vs. 601 in WT; *p* = 0.009; Figure 2B). The ratios of b-wave to a-wave amplitude for the three intensities were similar in KO and WT (ranged from 1.7 in WT and 1.8 in KO for the lower intensity to 3.6 and 3.7 for the higher intensity).

To determine if the reduced a-wave was due to compromised photoreceptor function, we computed the Lamb and Pugh amplification constant “*A.*” This constant provides a measure of the gain of the photoreceptor transduction cascade that is independent of light intensity for low-to-moderate intensities (Lyubarsky and Pugh, 1996). The amplification constant *A* was computed by fitting the a-wave component of two flash intensities (460 and 1860 R^*^/rod) to Equation 1 (Figure 2C; the fit is shown with dashed lines). The amplification constant *A* in WT (3.5) was not statistically different from that in KO mice (4.3; Figure 2D; *p* = 0.11), indicating that deletion of *PDE9A* does not affect phototransduction. Next, we looked at the shape of the rod/cone mixed response and computed the 66% maximum width. As expected, in both WT and KO, the rise time of the b-wave decreased slightly and the fall time increased substantially with increasing intensity. The curves of the rise time as a function of intensity for KO and WT were very similar. The curves of the fall time and 66% maximum duration for KO crossed those of WT: at lower intensities KOs were slightly faster, and at the highest intensity tested, the KOs took longer to recover (Figure 2E). However, neither of these differences was significantly different between WT and KO.

### Absence of PDE9A slows down the photopic response

**Figure 3:**
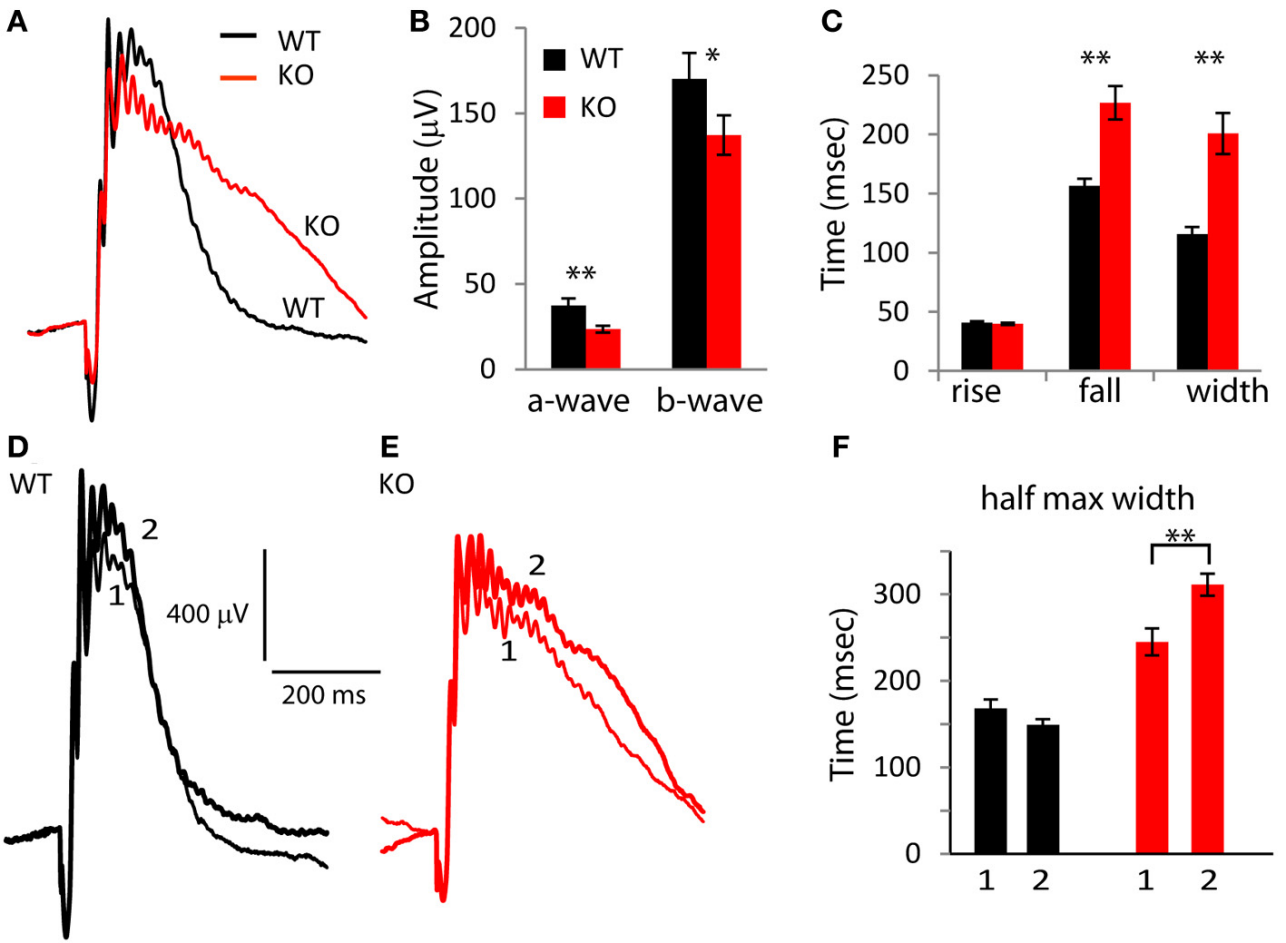
**Absence of PDE9A slows the falling phase of the b-wave of a saturating flash under photopic conditions. (A)** Average responses of WT and KO retinas to a saturating flash (1000 scot cd s m^−2^) after 2 s light adaptation. The responses to both the first and second saturating flashes (presented before and after stimulation with UV and green light, respectively) in each retina were averaged. **(B)** Response amplitudes of the a- and b-waves at the saturating flash are reduced in KO (13 WT and 14 KO mice). **(C)** The times taken by the b-wave to rise and fall to 66% of its amplitude and the duration between them. **(D,E)** Average response of WT and KO retinas to the first (1) and second (2) saturating flash. **(F)** The width of the b-wave at 50% of its amplitude for the first (1) and second (2) saturating flashes increased in KO.

To test the effect of deleting PDE9A on the photopic ERG wave, we suppressed rod responses using a light step of 30 scotopic cd m^−2^ and superimposed (in sequence, with 5.5 s delay between trials) a saturating white flash of 1000 scot cd s m^−2^, UV flashes (λ = 365 nm; 4 × 10^3^, 8 × 10^3^, and 1.65 × 10^4^ photon/μm^2^), green flashes (λ = 513 nm; 8 × 10^3^, 1.6 × 10^4^, and 3.2 × 10^4^ photon/μm^2^) and another saturating white flash of 1000 scot cd s m^−2^. In light-adapted rodents, the ERG a-wave is small and it reflects the activity of cones and the OFF pathway; the b-wave predominantly reflects the activity of ON cone bipolar cells and to a smaller extent that of the inner retina (Xu et al., 2003; Sharma et al., 2005; Shirato et al., 2008). For the saturated flashes, the average WT a-wave (combining the amplitude of both the first and the last flash) was 37.3 μV and in KO mice, it was 23.5 μV; significantly smaller than in WT (*p* = 0.004). The WT b-wave (170 μV) was larger than that of KO (137 μV) by 20% (albeit not significantly, *p* = 0.09; Figures 3A,B). Thus, absence of PDE9A greatly reduced the photopic a-wave amplitude, and only mildly effected the b-wave. Examination of the wave shape (Figure 3C) showed that while the times for the b-wave to rise to 66% of the peak in KO (40 ms) were similar to those in WT (41 ms) (*p* = 0.56), the time it took the b-wave to fall to 66% of its peak amplitude was highly significantly longer in KO (227 in KO vs. 156 ms in WT; *p* = 0.0001). Consequently, the 66% maximum width was also highly significantly longer in KO (201 in KO vs. 116 ms in WT; *p* = 0.0001).

Because photopic ERG responses were slower in KO than in WT, we wondered if light adaptation contributed to this difference. We therefore compared the first saturating response to the last saturating response in each experiment. Recall that the last saturating flash was presented after the UV and green flashes, so the eyes were exposed to the adapting light step for 12 min longer (Figures 3D–F). In WT, the kinetics of the b-wave in the first and last saturating flashes were similar, while in KO, the second presentation of the saturating flash resulted in a slower decay. The difference in the 66% maximum width was not significant (paired *t*-test; *p* = 0.1), but the 50% maximum width in KO was significantly longer in the second saturating flash (*p* = 0.007; Figure 3F). This suggests that PDE9A contributes to the kinetics of the cone-generated response, and that this contribution may be affected by the state of light adaptation.

**Figure 4:**
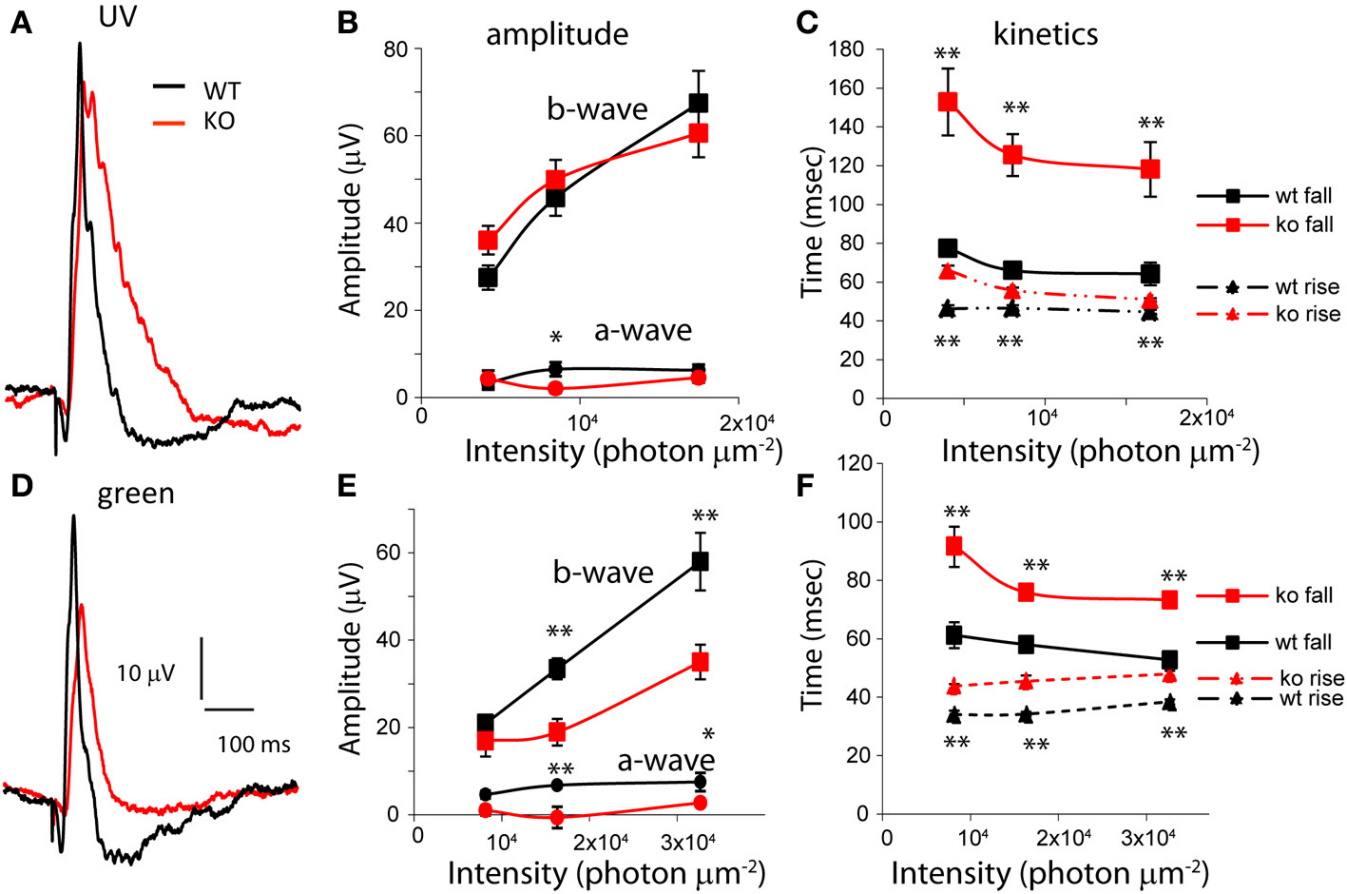
**PDE9A accelerates the kinetics of the photopic b-wave. (A)** Average responses from WT and KO retinas to ultra-violet (UV) light of intensity 1.65 × 10^4^ photons μm^−2^ (17 records were averaged for WT and 24 for KO). **(B)** Amplitudes of the a- and b-waves from WT (*n* = 9–12 mice) and KO (*n* = 12–13) retinas at increasing intensities of UV light. **(C)** The time taken by the b-wave to rise and fall to 66% of its amplitude at different intensities of stimulation. The time taken by the b-wave to rise and decay to 66% of its amplitude was significantly longer in KO at all intensities. **(D)** Average response of WT and KO retinas to green light of intensity 3.2 × 10^4^ photons μm^−2^ (*n* = 16 for WT and 19 for KO). **(E)** Amplitudes of the a- and b-waves from WT (*n* = 7–11) and KO (*n* = 2–11). Mice at increasing intensities of green light. **(F)** The times taken by the b-wave to rise and fall to 66% of its amplitude: both parameters are significantly longer in KO.

To see if the effect of PDE9A’s absence is specific to high intensities, or perhaps to different cone pathways, we examined the responses to low intensities of UV and green flashes. In response to UV flashes, both a- and b-waves amplitudes were similar in KO and WT (Figures 4A,B). However, in response to green flashes, both a- and b-wave amplitudes were significantly smaller in KO (Figures 4D,E). Like the saturating response, the time for the b-wave to decay to 66% and the 66% maximum duration were significantly longer for both wavelengths at all intensities (*p* < 0.01; Figures 4C,F). In addition, at these wavelengths, the time to rise to 66% of the peak amplitude was also significantly longer (*p* < 0.001). Consequently, the time to peak in KO for all intensities at both wavelengths was also significantly longer. Taken together, our ERG results suggest that the main effect of deleting PDE9A is on the photopic ERG which reflects activity in the cone pathways.

### PDE9A is localized to cells in the ganglion and amacrine cell layers

**Figure 5:**
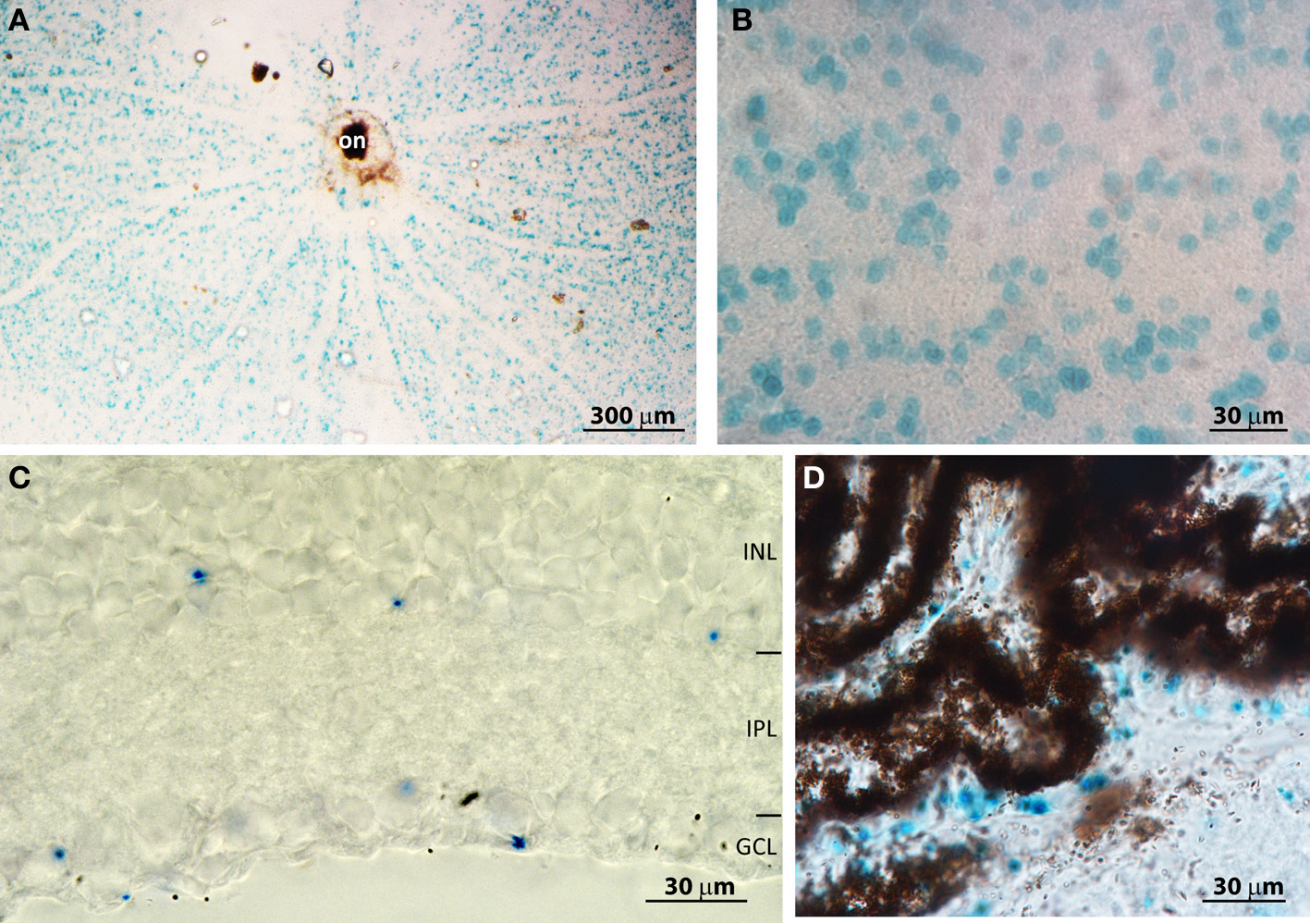
**PDE9A is localized in the inner retina. (A)** Low magnification (4×) image of a whole mount *PDE9A* KO retina stained for X-gal (blue); on, optic nerve head. **(B)** Higher magnification (40×) image of the ganglion cell layer (GCL) in the same retina as in **(A)**: the stained cells occupy less than 50% of the area. **(C)** A cryosection of a KO retina stained for X-gal. X-gal staining is present in both the inner nuclear layer (INL) and GCL. IPL: inner plexiform layer. **(D)** Cryosection of KO retina showing X-gal staining in the ciliary body.

To understand the mechanism by which PDE9A affects retinal processing, it is essential to determine its cellular localization. We initially attempted to localize PDE9A using two commercial antibodies; both gave intense staining in several layers of the retina. However, when the antibodies were applied to KO retinas, the staining patterns were similar to those of WT, suggesting that the antibodies cross-react with other proteins (not shown). We similarly obtained non-specific bands using Western blots with these antibodies. To get a reliable localization pattern, we stained retinas for the reporter *Lac-Z* that replaced *PDE9A* in KO. We first attempted to immunostain for β-gal, but there was no specific staining; instead we used the X-gal assay. In whole mount KO retinas, staining was present throughout the ganglion cell layer (GCL) (Figures 5A,B). No staining was found in WT, suggesting that this staining is specific and is present only in cells that normally express PDE9A. We determined the percentage of PDE9A-expressing cells within the GCL by co-staining the retina with DAPI. In a total GCL area of 150,000 μm^2^ (3 retinas combined) we found 1844 somas of which 28% were stained for X-gal. We also performed X-gal staining on vertical cryosections of the retina. Most staining was observed in cells of the GCL, but some staining was also observed in the somas of amacrine cells in the inner nuclear layer (Figure 5C).

Based on transcript presence and pharmacological blocking of certain PDE isoforms, it was suggested that retinal pigment epithelium (RPE) expresses PDE9A (Diederen et al., 2007). If so, absence of PDE9A from these cells may be able to explain the slow b-wave. We have therefore examined retinal cryosections that had RPE and choroid layers intact. There was no X-gal staining in the RPE; however we observed staining in the non-pigmented epithelium of the ciliary body (Figure 5D), in certain unidentified cells in the sclera, and in the lens (not shown). We further examined whole mounts of the pigment epithelium layer, but did not observe any staining.

**Figure 6:**
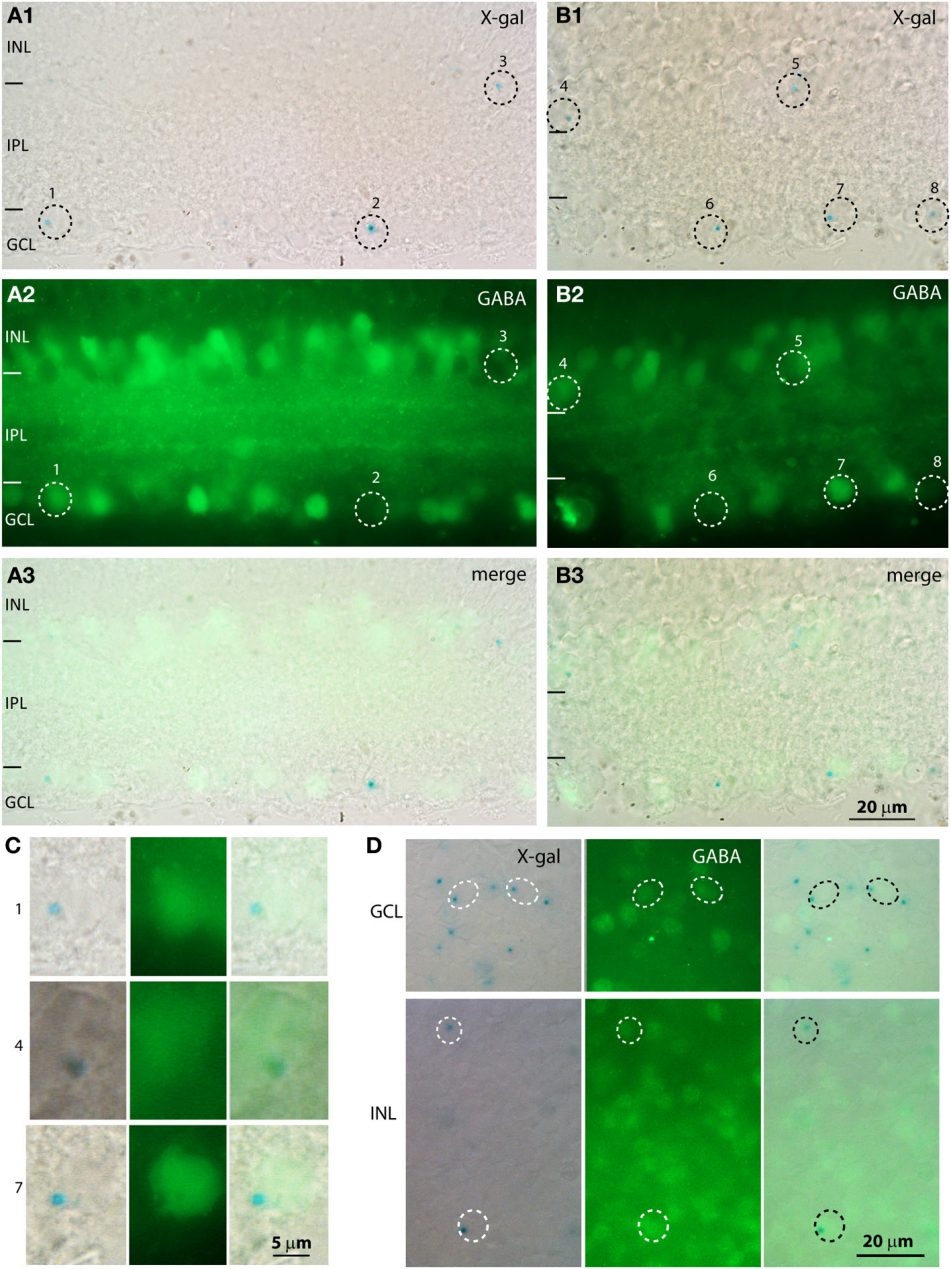
**A population of X-gal positive cells is positive for GABA. (A,B)** Cryosections from KO retinas double stained with X-gal (**A_1_,B_1_;** blue, visualized with DIC microscopy) and for GABA (**A_2_,B_2_**, visualized with fluorescence microscopy). Dotted circles indicate the location of the somas of the cells that are positive for X-gal. The merged images **(A_3_,B_3_)** show that some cells positive for X-gal are also positive for GABA and others are negative for GABA. **(C)** Magnified images of the X-gal positive cells (numbers on the side correspond to numbers on the low magnification images) that are also positive for GABA. Cells 1, 7 are located in the GCL and cell 4 is located in the INL. **(D)** Whole mount preparations stained with X-gal reagent and for GABA show that some cells in both GCL and INL stained for both (circles), while several X-gal stained cells were negative for GABA and numerous GABA-immunoreactive cells were not stained with X-gal.

To determine whether the cells in the GCL were amacrine or ganglion cells, we immunostained the X-gal stained retina for the amacrine cell markers GABA and glycine (Figure 6). Our antibody against glycine gave a high general background without specific staining, so the analysis relied on staining for GABA. We found that most X-gal-stained cells in the GCL were negative for GABA (e.g., cells 2, 6, 8 in Figures 6A–C), but many were positive (e.g., cells 1, 7). Similarly, in the inner nuclear layer, most X-gal-stained cells were negative for GABA but some were positive. To determine the percent of X-gal-stained cells that use GABA and the percent of GABA-immunoreactive cells that express β-gal, we co-stained whole mount retinas with X-gal and for GABA (Figure 6D). We found that in the GCL, 20% of X-gal-stained cells also stained for GABA, and 20% of GABAergic cells also stained with X-gal (3 retinas, Table 1). In the INL, 27% of X-gal-stained cells stained for GABA, but only 7% of the GABAergic cells stained with X-gal. This experiment suggests that PDE9A is present in one or more types of GABAergic amacrine cells, but mostly in GABA-negative cells.

**Table 1:** **Percentage of PDE9A-expressing cells in retina (averages of 3 retinas)**.

	**GCL**	**INL**
No. of X-gal-stained cells/206,000 μm^2^	495	219
No. of GABA-stained cells	325	509
% of X-gal cells that use GABA	20%	27%
% of GABA cells that expresses X-gal	20%	7%

## Discussion

We report here on two main findings. First, within the retina, PDE9A is restricted to the inner layers: it is widely distributed within the GCL and less widely within the amacrine tier of the inner nuclear layer. Second, PDE9A contributes to the amplitude and kinetics of the photopic ERG.

### PDE9A is localized to several retinal cell types

Our experiments identify at least three retinal cell types that express PDE9A: GABAergic amacrine cells, non-GABAergic amacrine cells (hence, glycinergic), and ganglion cells. The GABAergic amacrine cell type was identified by GABA-positive staining in a subset of X-gal-stained cells. We know that our immunostaining for GABA is genuine because we have previously shown that all cells that immunostain for GABA also express GAD_65_ or GAD_67_ (Vardi and Auerbach, 1995). In the inner retina, cGMP is predominantly synthesized by the soluble isoform of guanylate cyclase (sGC), which is activated by nitric oxide (NO). Immunostaining for the β 1 subunit of sGC (the obligatory subunit of the heterodimer in neurons) in rat retina shows that sGC is expressed in a subset of amacrine cells, some of which are GABAergic (Ding and Weinberg, 2007). Given that the retinas of rats and mice are sufficiently similar, it is likely that these cells may be the GABAergic PDE9A-expressing amacrine cells. It remains to be determined whether the GABA-stained amacrine cells in the GCL are of the same type as those in the INL (but displaced), or if they represent a different amacrine cell type. The glycinergic type is inferred because nearly all amacrine cells in the INL stain either for GABA or for glycine (Marc et al., 1995; Vardi and Auerbach, 1995; Kalloniatis et al., 1996), thus it is highly likely that the GABA-negative X-gal stained amacrine cells are glycinergic. This inference agrees with a previous report that NO-stimulated cGMP production is localized to glycinergic amacrine cells (Yu and Eldred, 2005). We think that one or more ganglion cell types express PDE9A because of the following facts: (1) about 40% of the GCL cells are ganglion cells (Jeon et al., 1998); (2) only few glycinergic amacrine cells are present in the GCL (Pourcho and Goebel, 1985, 1987; Marc et al., 1995); and (3) the PDE9A-expressing cells in the GCL are more numerous than those in the INL, in fact about 30% of cells in GCL express PDE9A (this study). This agrees with presence of *PDE9A* transcript in ganglion cells (Siegert et al., 2009) and with reports that NO stimulates production of cGMP in certain ganglion cells (Ahmad et al., 1994; Blute et al., 2003).

### PDE9A accelerates the photopic ERG b-wave by enhancing serial inhibitory responses

The most striking phenotype of deleting PDE9A is the change in the kinetics of the photopic b-wave. Under light-adapted conditions, at the tested UV, green, and saturating white light intensities, the falling phase of the b-wave in KO was slower, and the wave was wider than in WT. For UV and green flashes, the rising phase of the b-wave was also slower. Slower ERG b-wave in light-adapted conditions had been reported following intravitreal injection of TTX in mouse (Miura et al., 2009) and rat (Bui and Fortune, 2004; Mojumder et al., 2008). Similar effects of TTX on ERG kinetics were also reported in rabbit after 5 min of dark adaptation (Dong and Hare, 2000). Even a greater effect on the photpic ERG kinetics was found following injection of PDA (cis-2, 3-piperidinedicarboxylic acid which blocks transmission to hyperpolarizing 2nd order and all 3rd order neurons), or CNQX (Sharma et al., 2005; Miura et al., 2009). Another common qualitative outcome to the *PDE9A* KO and to applications of TTX, PDA, or CNQX is the reduced b-wave amplitude. While some of the effect of TTX in the light adapted retina is due to sodium channels in certain ON cone bipolar cells (Mojumder et al., 2008), an additional effect must be due to contribution from the inner retina, suggesting that spiking amacrine cells enhance and speed up the ON cone bipolar cells’ responses. The precise mechanisms by which amacrine cells affect the b-wave are not known. However, it is known that cone bipolar cells receive different types of inhibitory input from amacrine cells and that these amacrine cells themselves receive inhibitory input (serial inhibition). Furthermore, it has been shown by whole cell recordings and ERG recordings that these inhibitory circuits accelerate and often enhance bipolar cells’ responses (e.g., Dong and Hare, 2000, 2002; Molnar and Werblin, 2007; Eggers and Lukasiewicz, 2011; Eggers et al., 2013).

How can deletion of PDE9A mimic this effect? It is well known that many amacrine cells use cGMP to modulate their activity. For example, light stimulation increases NO levels in the IPL (Eldred and Blute, 2005) and this increases the levels of cGMP. NOS1 (the enzyme that produces NO in neurons) is expressed in certain types of GABAergic amacrine cells, and NO stimulates the production of cGMP in neighboring sGC expressing cells (Ding and Weinberg, 2007). It is possible that these sGC expressing amacrine cells are those that express PDE9A, and that these two enzymes regulate cGMP in a light-dependent manner. Since cGMP activates cGMP-dependent kinases (such as PKG), and since phosphorylation crucially controls exocytosis and endocytosis (Liu, 1997), absence of PDE9A can alter neurotransmiter release. Thus, if PDE9A-expressing cells inhibit bipolar cells in a time-dependent manner to regulate their response size and kinetics; absence of PDE9A, which likely causes an accumulation of cGMP, would render bipolar cells’ responses slower. Based on the characteristics of the b-wave seen in the absence of PDE9A and their resemblance to the pharmacological results described above, we predict that these cells spike at photopic intensities. Finally, although PDE9A is likely present also in ganglion cells, we do not think that they contribute to reducing the ERG b-wave and slowing down its kinetics because in WT, ganglion cells do not contribute to the ERG a- and b- waves (Bui and Fortune, 2004; Li et al., 2005; Mojumder et al., 2008). Ganglion cells, however, do contribute to the photopic negative response (PhNR) (Li et al., 2005), and although not quantified here, this wave might have been affected.

### PDE9A likely reduces the cone-generated a-wave due to an effect on the off pathway

We found that in the absence of PDE9A, the photopic a-wave elicited by both green and saturating flashes was smaller. In rodents, the photopic a-wave is generated by the activity of cones and post-receptoral neurons, which includes a small contribution from OFF bipolar cells and horizontal, and perhaps a larger contribution from amacrine cells (Xu et al., 2003; Sharma et al., 2005; Mojumder et al., 2008; Shirato et al., 2008). We think that the reduced a-wave in KO represents reduced current contribution from the inner retinal cells and not reduced cone activity because cones do not express PDE9A. Thus, the same disrupted signal processing that explains the reduced ON bipolar cells’ activity, as reflected by the ERG b-wave in KO, could explain the reduced inner retina component of the photopic a-wave (either an indirect influence on OFF bipolar cells’ activity or reduced current from amacrine cells). This interpretation also explains why the a-wave of the mix rod-cone ERG was smaller in KO than in WT retina. With such bright flashes, both cones and inner retinal cells likely contribute to the negative a-wave. In contrast, rod function has not been altered, as can be inferred from both the similar Lamb and Pugh amplification constants and the similar scotopic responses in KO and WT.

## Conclusion

The results of our study indicate that PDE9A, by hydrolyzing cGMP, controls its levels and thereby modulates inhibitory processes in the retina. We speculate that light increases nitric oxide levels in the cone pathway of the inner retina; this stimulates sGCs, increases cGMP levels, and modulates inhibition. By hydrolyzing cGMP, PDE9A restricts the duration of the inhibitory processes, thus sharpening and accelerating retinal signaling.

### Conflict of interest statement

The authors declare that the research was conducted in the absence of any commercial or financial relationships that could be construed as a potential conflict of interest.
